# The ram cichlid (*Mikrogeophagus ramirezi*) learns an associative task: a new fish species for memory research

**DOI:** 10.1038/s41598-023-40739-9

**Published:** 2023-08-23

**Authors:** Benjamin Tsang, Veronica Venditti, Celina Micaela Javier, Robert Gerlai

**Affiliations:** 1 Cell and System Biology Department, University of Toronto, Toronto, ON Canada; 2 Department of Psychology, University of Toronto Mississauga, Mississauga, ON Canada; 3 Department of Psychology, University of Toronto Mississauga, Rm CCT4004, 3359 Mississauga Road, Mississauga, ON L5L 1C6 Canada

**Keywords:** Learning and memory, Classical conditioning

## Abstract

Fish are the most species rich and evolutionarily oldest vertebrate taxon. This represents opportunities for biologists who intend to employ laboratory animals in their comparative or translational research. Yet, the overwhelming majority of such studies use a single fish species, the zebrafish, a suboptimal strategy from the comparative standpoint. Neuronal plasticity (learning and memory) is perhaps one of the most complex biological phenomena from a mechanistic standpoint, and thus its analysis could benefit from the use of evolutionarily ancient and simple vertebrate model organisms, i.e., fish species. However, learning & memory research with the zebrafish has been replete with problems. Here, we employ a novel fish species, the ram cichlid, we argue will be particularly appropriate for this purpose for practical as well as ethological/ecological reasons. First, we investigate whether the ram cichlid exhibits innate preference for certain colours (red, blue, yellow or green) in a four-choice task, the plus maze. Subsequently, we pair the apparently least preferred colour (green, the conditioned stimulus or CS) with food reward (the unconditioned stimulus, US) in the plus maze, a CS–US associative learning task. After eight pairing trials, we run a probe trial during which only the CS is presented. At this trial, we find significant preference to the CS, i.e., acquisition of memory of CS–US association. We argue that our proof-of-concept study demonstrating fast acquisition of CS–US association in the ram cichlid, coupled with the universal utility of some genome editing methods, will facilitate the mechanistic analysis of learning and memory.

## Introduction

Learning and memory, the two phases of cognitive processes that allow organisms to change their behaviour as a result of prior experience, are ubiquitous in the animal kingdom^1^. Learning and memory also play crucial roles in our own lives, and there are numerous brain disorders associated with abnormalities in these cognitive phenomena^2–4^. Thus, a large number of laboratories investigate the mechanisms of learning and memory and attempt to model human brain disorders with memory dysfunction using laboratory organisms. Most of these studies employ rodents^5^, but zebrafish have also started to be utilized^6,7^. The argument for the latter is that fish are the simplest and evolutionarily oldest vertebrates, thus, they may allow one to discover fundamental, core, mechanisms underlying the biological phenomenon of interest^8–10^. Thus, some learning and memory studies have already been conducted with the zebrafish (for reviews see^7,11–14^). But the zebrafish is not the only fish species with which learning and memory or other cognitive phenomena have been analyzed. In fact, there is a rich history of studies on fish cognition. For example, African cichlids have been studied for neophobia and learning performance^15^, and spatial learning has been demonstrated in other cichlid species^16^. The effect of social environment on learning has also been studied in cichlids^17^. Guppies have been used to test learning and memory and underlying neuroplastic changes in the brain^18^ as well as for brain morphology correlates^19^. The freshwater angelfish, a cichlid species, was shown to be able to remember the mental representation of larger versus smaller quantities of items^20^. Finally, mosquito fish has been shown to exhibit sex differences in cognitive performance^21^. Similar examples abound in the literature.

Zebrafish have been shown to be capable of simple CS–US (conditioned stimulus–unconditioned stimulus) associative learning^11–14,22,23^ and even more complex forms of associative learning, including spatial (relational) learning^24–26^. Although the overwhelming majority of studies with fish employ zebrafish, and the above studies have already demonstrated good learning performance in this fish species, the zebrafish may not be the best species among fish for learning and memory research. Could the ram cichlid, the subject of the current study address the shortcomings of the zebrafish? And is it better than other cichlids or other fish species employed in memory research? These are relevant questions to which for now we only have speculative answers. Before we discuss these speculations, however, we note that adding yet another fish species to the list of species employed in learning and memory research is expected to enhance the power of comparative approaches, and these comparative approaches are the studies that will enhance the translational relevance of our findings as well as better our understanding of the evolution of complex biological phenomena, including that of learning and memory^10^.

First, we briefly consider the possible advantages of the ram cichlid over the zebrafish in the context of learning and memory research. Some problems with zebrafish in the latter context have already been discussed^10^. Briefly, perhaps the most important among such problems is human handling. Human handling induces stress and fear responses, which represents a major confound^27–29^. For example, an immobile zebrafish hiding in the corner of the test tank is difficult to train. Another vexing issue concerns appetitive conditioning with food. This reinforcer, which is most frequently used with laboratory organisms in the analysis of learning and memory, has been rather problematic with zebrafish mostly because zebrafish satiate fast and also because we do not yet know what food types and how to deliver to this species^23^. An equally problematic aspect of zebrafish is their highly social nature. Most associative learning tasks require that the subject is trained individually, not in groups. Also, if one wants to follow the progression of acquisition of CS–US association across multiple trials and days, one must be able to distinguish each subject. As zebrafish look rather alike, and as individual marking of this small and delicate fish is an invasive procedure^30^, most often the experimenter must resort to isolating each subject until the completion of the experiment^14,22,31^. Isolation is also required during training and memory testing if one wants to avoid the complications associated with group learning. However, isolation for the highly social zebrafish has been found rather stressful^32,33^.

The ram cichlid is a small (4–5 cm long) freshwater fish from the tropical regions of South America (Venezuela and Colombia)^34^. From the aquarium hobby literature and from what we know about its natural habitat^35^, it appears to require similar water conditions (water chemistry and temperature) as the zebrafish^36^. The latter is a crucial consideration as established zebrafish facilities could easily adopt the ram cichlid without any modification to their operation and set up. In addition, unlike the zebrafish, the ram cichlid does not form shoals, and thus can be kept individually. Individual housing, as mentioned above, is a major advantage in learning and memory research as it allows the investigator to follow the change of learning performance across training and test trials of each and every subject without having to employ invasive marking techniques. Furthermore, unlike zebrafish, the ram cichlid moves slowly and thus its behavioural responses can be more easily tracked using commercial video-tracking systems as well as event recording-based methods^37^. Also notably, in nature and in the laboratory, the ram cichlid has been observed staying close to objects, moving into crevices or in between rocks^34^. Thus, unlike the zebrafish, it does not prefer open water and, we speculate, should be more comfortable in the confinement of mazes and other small tanks employed for learning and memory studies with fish. In its natural habitat, the ram cichlid swimming alone or in pairs (it is a pair bonding bi-parental species) may not attract aerial or large-bodied fish predators like zebrafish shoals would, and thus may not have robust antipredatory (fear and anxiety) responses towards human observers. This speculation is in line with our personal observations on lack of fear responses to the human experimenter moving around their tanks. Instead of avoidance reactions, we and others (anecdotical evidence from the aquarium hobby literature) observed ram cichlids to quickly learn when and where food is delivered to them by the experimenter, and respond positively by following the experimenter and gathering near the food delivery site. Last, we note that species that belong to the family Cichlidae are generally considered among the most “intelligent” fish species both in the aquarium hobby^38^ and in the scientific literature^39–41^. Although the ram cichlid has not been employed in learning studies, it is known to possess a sophisticated behavioural repertoire that implies good cognitive and mnemonic abilities^15–17,20,42,43^. For example, unlike the zebrafish, it is a bi-parental cichlid that raises its young, protects its territory, and stays in pairs, implying sophisticated intra-specific communication, spatial memory as well as recognition of individuals.

As cichlids are considered by aquarium hobbyists some of the most intelligent fish species and as there have been a variety of studies conducted with this family of fish species also suggesting good learning and memory performance^15,16,18,20^, why would we suggest the ram cichlid as an alternative better than previously employed cichlid or other fish species? The reasons overlap with those we discussed above when we briefly compared zebrafish and the ram cichlid. Most cichlids studied for their cognitive function has been either large-bodied South American cichlids, which although require similar water conditions the ram cichlid and the zebrafish do, could not be kept in the standard tanks of the zebrafish facility and would require significant investment for retooling. For the same reason, the number of zebrafish or ram cichlid that can be kept in a small laboratory could not be matched with these large cichlids. Another group of cichlids, the species from the great African lakes (Malawi, Tanganyika and Victoria) are usually about twice to three times the standard length of the ram cichlid, but their biggest drawback is that they require very different water chemistry, i.e., extremely hard and alkaline water, compared to what zebrafish facilities and what the ram cichlids require. Furthermore, given the large volume of these lakes and with it the highly stable water conditions, African cichlids are known in the aquarium hobby as well as in the scientific literature^44^ to be highly sensitive to water chemistry fluctuations as well as to accumulation of organic waste (e.g. ammonia, nitrite and nitrate). Briefly, for these practical reasons alone, the ram cichlid is a better choice. Different advantages may be speculated about when comparing the ram cichlid to live bearers, including poecilids, a group of fish into which the above mentioned guppy and mosquito fish belong. These species are highly social and would suffer from isolation induced stress. Furthermore, as live bearers, a single female only produces 10–12 fry about every 3–4 weeks, a number that is at least an order of magnitude less than what zebrafish and ram cichlids spawn.

For the above reasons, we propose that the ram cichlid may be a better subject of learning and memory research than the zebrafish or most other fish species employed in cognition research as of today. In this proof-of-concept study, we provide the first piece of evidence that the ram cichlid is indeed capable of acquisition of CS–US association, which we argue lays the foundation of further analyses of its cognitive capabilities. First, we examine whether the ram cichlid exhibits innate preference for particular colours. Next, using the apparently least preferred colour as the CS, we train the fish to associate it with food reward (the US). Last, in a probe trial, when no US is present, we demonstrate a significant preference for the CS, i.e., memory of the CS–US association.

## Materials and methods

### Animals

The parents of the experimental subjects were purchased from a local aquarium store, Big Al’s (Mississauga, ON, Canada). The offspring were raised, and at their age of 8 months (young adults) were used for the experiments. The sample size (n) was 10. 35L housing tanks (45 cm in length × 30 cm in height × 27 cm in width) were set up for the experimental fish in the Gerlai Zebrafish Facility (University of Toronto Mississauga, Ontario, Canada). The housing tanks were divided in the middle by a white corrugated plastic sheet, and a single experimental fish was kept in each half of the tank. White corrugated plastic sheets also covered the sides of the tanks so that the experimental fish could not see each other but could see out the front and back of the tanks. A sponge filter employing air bubbles was placed in each half-tank for filtration and oxygenation of the water. All water parameters (temperature = 25 °C, pH  7, Salinity = 300 μS, NH_4_OH = 0 ppm, NO_2_^−^ = 0 ppm, NO_3_^−^ = 0 ppm) were kept identical for all housing tanks (we emphasize that these parameters are also identical to what we employ for zebrafish in our facility). Light cycle was maintained by timers at 13 h light:11 h dark, with lights turning on at 7:00 h and off at 20:00 h. The experimental fish were fed bloodworms (pathogen free frozen larvae of midge flies belonging to the Chironomidae family by Hikari USA, Hayward, California) twice per day. The subjects were given two weeks to acclimate to the Zebrafish Facility before they were subjected to two different behavioural paradigms.

### Apparatus

**Figure 1:**
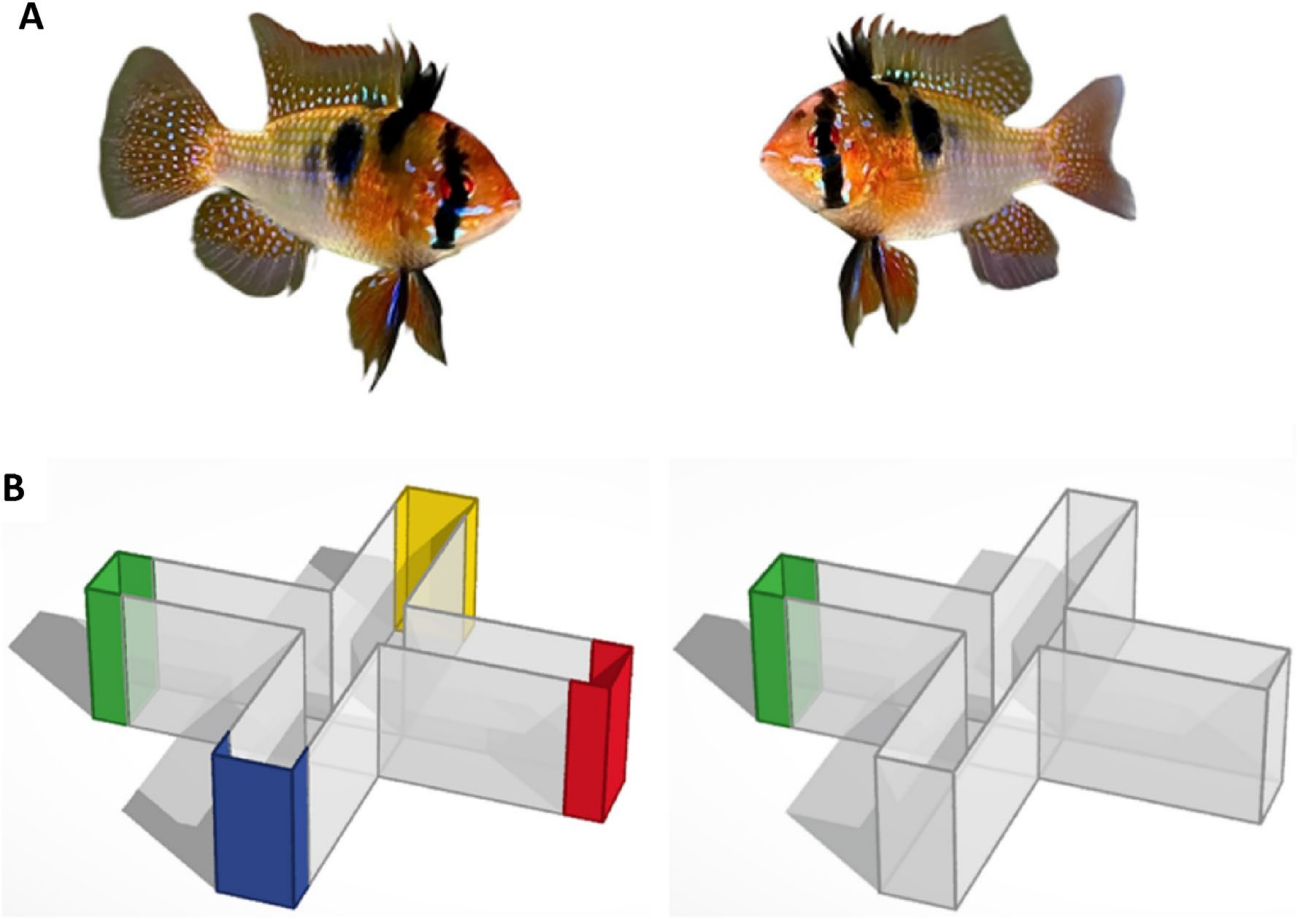
Pair of adult male ram cichlids (**A**) and computer rendering of the plus maze tank used in the colour preference and associative learning tasks (**B**). The tank consisted of four identical arms (length: 35.4 cm, width: 9.3 cm, height: 24.5 cm) and a square center region (length: 9.3 cm, width: 9.3 cm, height: 24.5 cm). During the colour preference experiment, red, yellow, green and blue construction paper was positioned at the end of each arm (**B** left). During the associative conditioning experiment, only green was used for one arm of the maze (**B** right). The perimeter of the plus maze was surrounded by a 1-m-high cardboard wall to increase environmental homogeneity throughout the experiment.

A plus-shaped maze was employed^14^ to examine both colour preference (Fig. 1B left) and associative learning (Fig. 1B right) in ram cichlids (Fig. 1A). All four of the maze's arms had the same dimensions (length: 35.4 cm, width: 9.3 cm, height: 24.5 cm). Construction paper of different colours (red, yellow, green, and blue for the colour preference test) was placed at the sides and bottom of each arm end so that the paper covered the entire wall of the end of the maze and extended 5.5 cm into the side walls (for training trials and the probe trial only the green color was employed) (Fig. 1B).

In the colour choice task (experiment 1) the colours were rotated clockwise after each fish was tested. For the associative conditioning task (experiment 2), a single colour, green was used by placing the green paper at the end of one of the four arms. The location of this green paper was also rotated across experimental fish. The plus-maze was surrounded by brown cardboard (1 m tall) to obstruct access to extra-maze visual cues, and thus to avoid spatial bias due to extra-maze visual cues, in both experiments.

### Procedure

#### Experiment 1: colour preference

First, we wanted to investigate whether ram cichlids exhibit spontaneous (prior training or instinctive) preference for particular colours, in order to eliminate the potential confound of colour bias in, and to find the least preferred colour for, the associative learning task (experiment 2). Four colour stimuli (blue, red, green, and yellow) were presented simultaneously in the plus maze as described above (Fig. 1B left). Before the experimental fish was put into the plus maze, three adult non-experimental ram cichlids (the stimulus fish) were placed into the plus-maze for 5 min in order to introduce ram cichlid olfactory cues into the maze and thus reduce potential stress upon introduction of the experimental fish. This protocol was repeated every trial for both experiment 1 and 2. Experimental fish were taken from their home tank using a small plastic container and added one at a time to the plus-maze. The experimental fish was then allowed to explore the maze for 10 min. This was repeated over four days, with two trials per subject (one in the morning and one in the evening) occurring each day, a total of 8 trials. The location of the coloured ends was rotated clockwise each day to avoid spatial bias. Experimental tank water was identical in temperature and chemistry to the water of holding tanks and was changed after every trial. The last colour choice trial was video-recorded and the recordings were later analyzed.

#### Experiment 2: associative conditioning (CS–US association training and probe)

In the first experiment, we found no significant colour choice bias by ram cichlids. Nevertheless, an apparent lower preference for green compared to the other colours was seen. Thus, we decided to use this apparently least favoured colour as the CS in this next experiment, the associative learning task (Fig. 1B right), and we paired this CS with food (US) as described below. The food we employed has been known to be a favourite of ram cichlids in the aquarium hobby: the bloodworm (larvae of the fly, *Glycera dibranchiata*).

### Habituation

The fish used in this experiment were the same as those tested in the first experiment (n = 10). Experiment 2 took place a month after the completion of experiment 1, i.e., each experimental fish had a 30-day-long inter-experiment break. All experimental fish were first re-habituated to the plus-maze by placing them one at a time into the maze with no colours showing at the end of the arms, and no food being present in the maze. Habituation took place over two days, with each subject being allowed to explore the maze for two 10-min intervals per day (one in the morning and one in the evening) for a total of four occasions.

### Training

Next, subjects underwent four days of training. The plus-maze had three uncoloured arm ends and one green arm end. Approximately 10 mL of bloodworm water was evenly distributed in the maze. The bloodworm water was made by mixing three blocks of bloodworms (1 block is a frozen cube of 3.4 cm^3^ volume Hikari, Bio-Pure Blood Worms) with 30 mL of RO water. Subsequently, the solution was allowed to stand for 15 min, the supernatant was removed and was used as bloodworm water. This ensured that the water in the maze had olfactory cues from ram cichlids and also that it had a homogeneously distributed olfactory cue indicating the presence of bloodworms, a smell of food that masked the actual location of the food reward. Fifteen bloodworms, the US, were placed at the end of the arm of the maze with the green paper, the CS. We note that the bloodworms had sunk to the bottom of the tank and remained there. Experimental fish were moved from their home tank in a small container, and were released into the maze singly by immersing the container in the center of the maze. The experimental fish was allowed to freely explore the maze for 10 min during which the experimenter left the room. Training occurred over 4 days, with two 10-min sessions per day (one in the morning and the other in the evening) for each experimental fish. Each day, the location of the CS/US was changed to eliminate potential spatial bias. During the training period, no other food was given to the cichlids, i.e., feeding stopped in their home tank.

### Probe

After the completion of the eight training trials, on the next day immediately following the last day of training, a memory probe trial was conducted. During this trial, only the CS (the green colour cue) was presented, and no US (food reward) was administered. The arm end where the green colour cue was presented rotated across experimental fish. The probe trial, just like the training trials, was 10 min long, and all other methodological aspects were also identical to those of the training trials.

### Quantification of behaviour

Behaviour of the experimental fish was recorded using a video camera (HDR-CX405; Sony) mounted above the plus-maze. Video recordings of the last colour choice trial of Experiment 1 and of the probe trial of Experiment 2 were transferred to a PC (XPS Desktop, Dell) and later analyzed using Ethovision XT 14.0 (Noldus Info Tech, Wageningen, The Netherlands). The default Ethovision detection settings were modified to more efficiently detect each subject [video-frame rate = 25; method = dynamic subtraction; subject colour compared to background = darker (18–225); frame weight = 2; subject size = 250–500 pixels], and then each video was separately acquired. Each subject’s location was tracked automatically by the Ethovision software’s Track Editor tool using center-point detection, and rare mistakes in tracking (e.g., track spikes due to bubbles or floating debris) were manually corrected. We quantified the duration of time the experimental fish spent in each end zone. End zones, the size of the coloured maze-arm ends, were marked in the analysis software. End zones of the same size were also marked for arm ends with no colour cues present (3 arms of the maze in Experiment 2). A subject was considered to have entered a given end zone once its center-point crossed the zone threshold. Motor patterns, i.e., swim path parameters of the ram cichlid have not been described. Thus, to characterize general motor activity, we quantified swim speed (or distance moved per unit of time, in cm/sec), turn angle (measured in degrees, turn angle is the absolute angle of a change in the direction of movement from one frame to the next; direction of the turn (clockwise or counter-clockwise) was not considered), duration of immobility (or freezing, quantified as the amount of time in seconds in which less than 20% of the pixels composing the body area of each subject changed between video frames) and as well as the intra-individual temporal variance of the former two measures and the frequency of the last measure. We quantified these motor responses for the last colour choice and the probe trial^37^.

### Statistical analysis

Data were analyzed using IBM SPSS (version 24) statistical software package written for the PC. First, we investigated general motor patterns, swim speed, turn angle and immobility and whether fish behaved differently between the last colour choice test (experiment 1) and the probe trial (experiment 2) to obtain a general description of the swim path parameters of the experimental ram cichlids. We plotted these path parameters as a function of 30 s long time-intervals and employed nested repeated measures ANOVA to study whether interval (within subject factor 1 with 20 levels, the 20 thirty-sec long intervals) and trial (within subject factor 2 with 2 levels (colour choice trial, probe trial)) had a significant effect. As swim path parameters of the ram cichlid have not been described or quantified, these results will form a baseline for future studies. Next, we employed a repeated measures ANOVA to investigate whether there was any innate colour preference by the ram cichlid in the 4-colour choice test during the last colour choice trial (experiment 1), with colour zone as the within subject factor (4 levels: blue, green, red, yellow). Last, and most importantly, we investigated whether ram cichlids acquired the association between CS and US, i.e., whether they spent more time in the proximity of the CS during the probe trial. To test this, we compared time spent in the green zone with the average of time spent in the three other end zones of the plus-maze using one-tailed paired sample t-test. In addition, we also investigated whether the experimental fish spent more time in the green zone during the probe trial (i.e., after training) than in the green zone during the colour choice test (i.e., before training) using one-tailed paired sample t-test.

### Ethical declarations

All experimental protocols were approved by Local Animal Care Committee at the University of Toronto Mississauga in accordance with LACC and CCAC guidelines and regulations. All methods are in accordance with ARRIVE guidelines.

## Results

**Figure 2:**
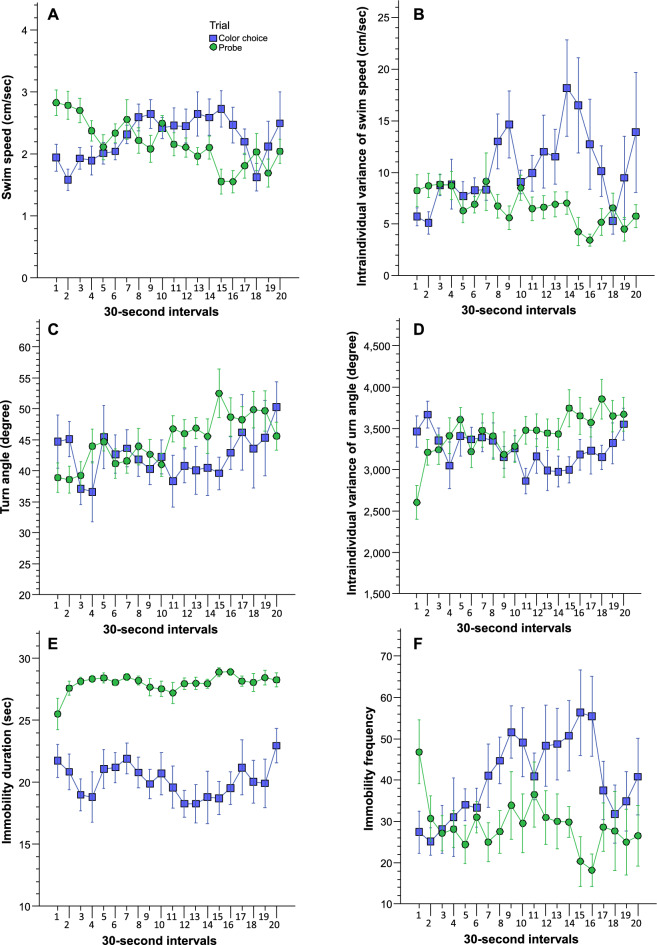
Swim path parameters (swim speed (**A**); intraindividual temporal variance of swim speed (**B**); turn angle (**C**), intraindividual temporal variance of turn angle (**D**); immobility duration (**E**); and Immobility frequency (**F**) recorded during the last colour choice trial, i.e., before training (blue rectangles) and the probe trial (green circles), i.e., after training are shown as a function of 30-s time intervals. Means ± S.E.M. are shown. For statistical analysis results, see “Results”.

Experimental ram cichlids appeared active and explored the maze during both the last colour choice trial and the probe trial a month later. Their movement pattern appeared different from that of zebrafish as they swam more slowly and interrupted their locomotion with frequent stops and turns^45,46^. Furthermore, the motor responses of ram cichlids also appeared different between the colour choice trial and the probe trial. These subjective observations are supported by the analysis of video-tracking extracted swim path parameters (Fig. 2). Analysis of swim speed (Fig. 2A) suggested that during the colour choice trial swim speed gradually increased during the first two thirds of the trial, while it decreased during the probe trial. Nested repeated measures ANOVA confirmed this observation and showed that the effects of trial (F(1, 9) = 0.393, p = 0.546) and of interval (F(1, 19) = 1.533, p = 0.08) were non-significant, but the interaction between these factors was significant (F(19, 171) = 4.336, p < 0.001). Intraindividual temporal variance of swim speed (Fig. 2B) also appeared to show a trial specific temporal trajectory: it appeared to increase with some variation throughout the colour choice trial, while it remained fairly stable and showing decreasing values with time during the probe trial. ANOVA showed that the effect of trial was significant (F(1, 9) = 10.001, p = 0.012), the effect of interval was non-significant (F(1, 19) = 1.167, p = 0.291), and the interaction between these factors was significant (F(19, 171) = 2.271, p = 0.003). Unlike swim speed and intraindividual temporal variability of swim speed, turn angle (Fig. 2C) did not appear to change between the colour choice and the probe trial, and also did not appear to change across the 30-s time intervals of these trials. ANOVA found no significant effects of the main factors (trial, F(1, 9) = 3.073, p = 0.114; interval (F(1, 19) = 1.537, p = 0.078), and the interaction term was also non-significant (F(19, 171) = 1.223, p = 0.244). Intra-individual temporal variance of turn angle (Fig. 2D) appeared to change across the 30-s time intervals in a trial dependent manner. ANOVA confirmed this observation and showed that the effects of trial (F(1, 9) = 2.455, p = 0.152) and of interval (F(1, 19) = 1.552, p = 0.074) were non-significant, but the interaction between these factors was significant (F(19, 171) = 2.138, p = 0.006). Duration of immobility (Fig. 2E) showed a robust difference between the colour choice and the probe trial: during the colour choice trial, ram cichlids were immobile for much shorter duration of time compared to during the probe trial. ANOVA confirmed this and demonstrated a highly significant trial effect (F(1, 9) = 338.98, p < 0.001), but found no significant interval effect (F(1, 19) = 0.764, p = 0.746) or interval × trial interaction (F(19, 171) = 0.945, p = 0.528). The frequency of immobility episodes showed a different pattern of results (Fig. 2F). ANOVA found the effects of trial (F(1, 9) = 4.085, p = 0.074) and of interval non-significant (F(1, 19) = 1.291, p = 0.195). But the interval x trial interaction was significant (F(19, 171) = 2.970, p < 0.001). In summary, ram cichlids were active, moved with slow speed, turned and stopped frequently, and explored the entire maze.

**Figure 3:**
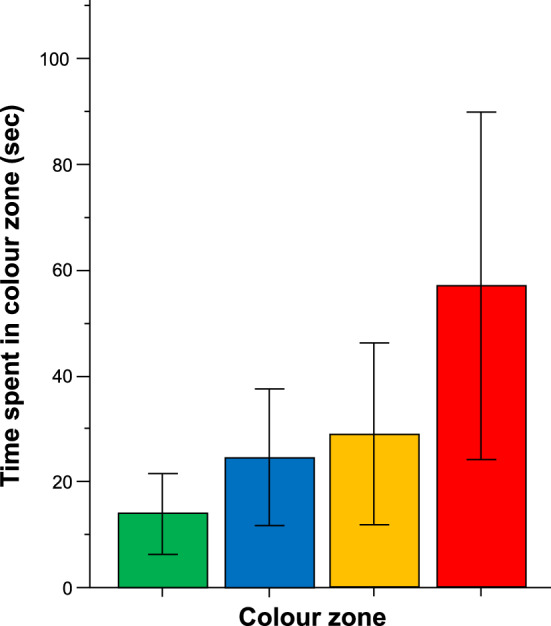
Time spent in the end-zones of a plus maze, each marked by a different colour (green, blue, yellow and red). Means ± S.E.M. are shown. Note that the time experimental ram cichlids spent in the different colour marked end-zones was found not to differ significantly. For detailed statistical analysis results, see “Results”.

After the above characterization of swim path parameters of the ram cichlids during the last colour choice trial and the probe trial, we examined whether the fish showed any innate (non-reinforced, spontaneous) preference for any of the four colours presented during the last colour choice trial. Although experimental ram cichlids appeared to spend different amounts of time in the end zones marked by the four distinct colours (Fig. 3), ANOVA did not confirm this observation as it found no significant colour zone effect (F(3, 27) = 0.725, p = 0.546). Nevertheless, as green appeared to be the least preferred colour by the experimental fish, we decided to use this colour as the CS for the subsequent CS–US conditioning trials, and paired it with reward, i.e., with food during the training trials. During training, the food is presented together with the green colour cue. Thus, training trial performance may reflect the direct effect of food, not acquisition of memory. Thus, we do not present training trial performance here, and instead, focus our analysis on the probe trial, during which only the CS, the colour cue, may guide the experimental fish (as the food reward, the US, is absent).

**Figure 4:**
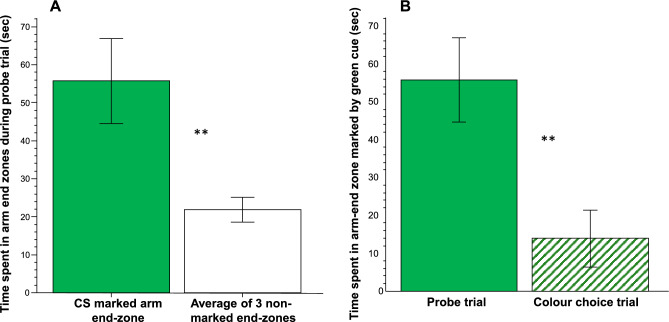
During the probe trial, i.e., after CS–US conditioning, ram cichlids spend significantly more time in the end-zone of the arm of a plus maze marked by the CS (green colour) compared to how much time they spend on average in the (non-marked) end-zones of the other three arms (**A**). Ram cichlids also spend significantly more time in the CS (green) marked end-zone during the probe trial, i.e., after training, compared to how much time they spend in the green-marked end-zone during the colour choice trial (**B**). Means ± S.E.M. are shown. Significant (p ≤ 0.01) differences are indicated by **. Note that during the probe trial, US (food) is absent, and only the CS (green cue) is presented. For detailed statistical analysis results, see “Results”.

Analysis of the probe trial performance thus allows us to investigate whether the experimental ram cichlids have developed a preference for the green colour as a result of their training (co-presentation of this colour and food during the eight training trials). Significant preference for the green coloured zone would demonstrate acquisition of memory of the association between the CS (the green colour) and the US (food). Figure 4A shows the result of the probe trial conducted 24 h after 8 CS–US pairing trials. The figure suggests that the experimental fish spent more time in the end-zone marked with the green paper compared to the average of the time they spent in the three other end-zones not marked by any colour, an effect that was found significant by paired t-test (t = 3.052, df = 9, p = 0.007). Furthermore, Fig. 4B also shows that the amount of time the experimental fish spent in the green end-zone after training (i.e., in the probe trial) was more than the amount of time they spent in the green end-zone before training (i.e., during the colour choice test). Again, paired t-test demonstrated that this difference was significant (t = 2.833, df = 9, p = 0.01).

## Discussion

The movement patterns of ram cichlids have not been characterized before. Here, we decided to use video-tracking to quantify three important aspects of their swim path: swim speed, turn angle and immobility. Although we did not systematically compare ram cichlid motor performance with zebrafish motor performance using a randomized controlled analysis, for reference, below we discuss what the typical values of certain swim path parameters have been found in the past for zebrafish under circumstances similar to those of the current study, and how these values compare to the ram cichlid values we obtained here. Ram cichlids were found to swim with a speed between 1.5 and 2.8 cm/s. Whereas similarly sized adult zebrafish have been found to swim with a speed between 5 and 7 cm/s^28^ or even faster (5–10 cm/s), e.g., in a learning study^47^. Ram cichlids, unlike zebrafish, also stopped more frequently, stayed immobile longer and turned more, giving the subjective impression of a fish that explores its environment and attends to its features. For example, while in the current study we found ram cichlids to turn on average 40–50 degrees, and stay immobile for 20–28 s during 30 s intervals (i.e., for about 66–93% of the time), zebrafish under similar circumstances were found to turn only 7°–10° and remain immobile for 18 s/30 s interval (i.e., for 60% of the time)^46^. Intra-individual variance of speed we found lower for the ram cichlid (5–18 cm^2^/s^2^, compared to the zebrafish (25 cm^2^/s^2^)^46^, suggesting that the ram cichlids swam not only slower but this slower speed was more consistent than in zebrafish. Whereas we found ram cichlids to vary their turns more compared to zebrafish’s (intraindividual variance of turn angle we found to be 3200–3600 deg^2^ for the ram cichlids vs. 460 deg^2^ for the zebrafish)^46^. Last, frequency of immobility for the ram cichlid we found to be around 30–50 per 30 s interval, whereas it was found 0.5–1 per 30 s interval for the zebrafish^46^. These numbers confirmed the impression made by human observers that the ram cichlids appear to move more slowly, explore, and seem to attend to environmental cues better than the zebrafish, and thus appear better suited to the small experimental tanks of the laboratory than the zebrafish.

Comparison of swim path parameters recorded during the last colour choice trial (before training) and the probe trial (after training) also revealed some interesting findings. Ram cichlids showed elevated swim speed as well as elevated intraindividual temporal variance of swim speed during the colour choice trial as their trial progressed, but did the opposite, i.e., reduced their swim speed and variance of swim speed with time during the probe trial. A similar pattern of trial specific temporal changes was also evident for immobility frequency. These results suggest that ram cichlids moved with decreasing speed and increasingly more consistently with time during the probe trial perhaps because after the initial phase of exploration of the maze, they stayed in the proximity of the arm-end marked by the CS. Whereas they did the opposite in the colour choice trial (increased their speed and the variability of their movement) as they were exploring the maze with the four colours at the end of its arms. These observations are in line with the most robust difference found between the motor performance of ram cichlids recorded during the colour choice and the probe trial: during the latter, the fish stayed immobile much longer than during the former. This again suggests that in the probe trial they explored the plus maze much less, perhaps because they remained in the area (the end of the arm of the maze) marked by the CS. This speculation is supported by the analysis of how much time they spent in the end of the arm marked by CS versus other arm ends.

Briefly, we found adult ram cichlids to spend significantly more time in the end of the arm of a plus maze that was marked by green colour compared to the average time they spent in the other three arms that had no colour marking during the probe trial. This probe trial was conducted after only 8 training trials during which the green colour (CS) was co-presented with food reward (US). We emphasize that during the probe trial, no food reward was presented, and no odour cues could have guided the experimental fish. We also note that during the training trials as well as during the probe trial, the location of the CS was systematically rotated from arm to arm across the experimental subjects. Thus, alternative cues and strategies, e.g., extra-maze or intra-maze cues other than the CS, or ego-centric navigation could not have been utilized by the experimental fish. Importantly, we also found that during the probe trial, i.e., after the eight CS–US pairing training trials, experimental fish spent significantly more time in the green marked end-zone compared to how much time they spent in the green marked end-zone during the colour choice task, i.e., before training. Thus, we conclude that the experimental ram cichlids acquired, consolidated and recalled the CS–US association, i.e., they learned that the green colour predicted the presence of food. Given that the probe trial was conducted 24 h after the last training trial, we also conclude that the ram cichlids’ memory of the CS–US association lasted at least a day.

It is not surprising that the experimental ram cichlids were able to learn and remember the CS–US association. The plus maze and its procedure were designed to fulfill the requirements of associative conditioning. Consider that when the experimental fish were swimming in this maze, it first perceived the CS, as it was clearly visible from the entrance to the arm whose end was marked by the CS. When the fish entered the green end-zone, it encountered the food reward. Thus, the CS preceded and predicted the appearance/presence of the US. What is noteworthy, however, is that the ram cichlids could acquire the CS–US association within as few as eight training trials, and also that we needed only ten subjects to find this memory acquisition significant.

Zebrafish as well as other fish species have been found to acquire memory of CS–US association^11–13,16,17,22,40,48,49^. However, the studies in which such learning/memory performance was demonstrated with these fish species often required larger number of training trials and more subjects than employed in the current study. For example, in our own studies with zebrafish, we employed plus mazes and colour cues as CS and food as US, but we needed to conduct 20 training trials to find significant memory acquisition^13^. In another study of ours, we decided to employ a different US, as food reward turned out to be rather problematic. This US was the sight of conspecifics^50^. In this latter study too, we were able to prove acquisition, consolidation, retention and recall of CS–US association using the plus maze in zebrafish, but we needed 16 training trials. In another plus maze study, the first one in which we proposed and proved the effectiveness of the sight of conspecifics as reward^51^, we used 20 fish per training group to find significant memory consolidation. Yet in another associative conditioning task, also using the sight of conspecifics as reward and colour as the CS, we employed 20 training trials and 16 zebrafish per conditioning group^22^. In a shuttle box paradigm, we needed 35–50 zebrafish to find significant learning effects^52^. In summary, the current study with ram cichlids showed that significant CS–US acquisition can be achieved with fewer number of training trials and fewer number of subjects compared to those that employ zebrafish, the predominant fish species of biomedical and basic behavioural neuroscience research.

Nevertheless, we regard our study as only proof of a concept, i.e., the first indication that perhaps this new fish species, the ram cichlid, may be appropriate for learning and memory research. This is because systematic comparison of cognitive and mnemonic features and abilities of the zebrafish versus the ram cichlid has not been made. Even systematic analyses of the minimum number of trials needed to accomplish significant acquisition of CS–US association under different training conditions, for example, with different unconditioned and/or conditioned stimuli, in zebrafish is lacking. Nevertheless, the current proof-of-concept study does show that ram cichlids may be a promising study species for learning and memory research. A counterargument to this is that there are a large number of molecular and neuroscience methods developed or adopted for the zebrafish, and a newcomer like the ram cichlid is in a disadvantaged position from this standpoint. However, with the advent of modern genome editing methods, e.g., the CRISPR/Cas system^53^, which may be employed with a variety of species, we argue that this disadvantage is not substantial. Similarly, primarily due to evolutionary conservation, numerous other biology, including neuroscience and pharmacology, tools may be transferrable to the ram cichlid.

In conclusion, we propose that the ram cichlid has the potential to become an excellent model organism for the analysis of cognitive and mnemonic functions of vertebrates, allowing a simple yet efficient way to discover behavioural aspects of complex brain functions and to study underlying mechanisms.

## Data Availability

The datasets used and/or analysed during the current study available from the corresponding author on reasonable request.
